# Advances in Fracture Healing Research

**DOI:** 10.3390/bioengineering11010067

**Published:** 2024-01-09

**Authors:** Sabrina Ehnert, Tina Histing

**Affiliations:** Department of Trauma and Reconstructive Surgery, Eberhard-Karls-University Tuebingen, BG Unfallklinik, 72076 Tuebingen, Germany; sehnert@bgu-tuebingen.de

Despite a constant refinement of surgical techniques and bone fixation methods, up to 15% of fractures result in impaired healing or even develop a non-union. The resulting immobility during convalescence strongly affects patients’ lives. In the past decades, many risk factors for delayed bone healing have been identified; however, the underlying mechanisms are still poorly understood, and the online monitoring of fracture healing is a great challenge. Therefore, this current Special Issue focused on recent advances in fracture healing research. This Special Issue comprises 13 original articles and 2 reviews covering a wide range of topics related to fracture healing.

Both review articles give a broad overview of the basic and clinical aspects of fracture healing. One of the review articles provides a general overview of the recent advances in animal models for studying bone fracture healing [1]. This review is complemented by an in vivo study describing a new model which combines surgical and pharmacological intervention to induce metaphyseal non-union in osteoporotic rats [2].

The second review systematically analyzed risk factors for pathological fracture healing and non-unions, providing the clinical background for the topic [3]. Two of the in vivo studies more closely investigated age as a risk factor for delayed fracture healing. While one study well-characterized age-dependent differences in callus formation and mineralization, which result in reduced mechanical strength following fracture healing [4], the second study additionally addressed the role of severe blood loss as an additional risk factor for impaired fracture healing [5]. One tool to investigate associated wound healing is given by another article describing a novel in vitro model simulating a scab [6]. An option for intervention is given by the ex vivo study in this Special Issue, which describes a novel periosteal-derived membrane with osteoinductive properties, addressing cell therapeutic strategies in fracture healing [7].

The use of growth factors or cytokines as therapeutics in fracture healing was also addressed. Bone morphogenetic protein 2 was used to re-activate bone remodeling in mice with the long-term and high-dose treatment of zoledronic acid [8].

Another aspect covers the mechanical stimulation of fracture healing, starting in vitro with a study showing anti-apoptotic effects of Focused Low-Intensity Pulsed Ultrasound (FLIPUS) on osteocytic cells, which opens up new perspectives as a non-invasive adjunct therapy promoting bone regeneration and repair [9]. These findings are complemented by an in vivo study showing positive effects of electric stimulation on critical size defects in rat calvaria filled with HA/TCP composite scaffolds [10].

Other studies focused more on the development, generation, and characterization of novel bone material to fill such large-size defects. For example, the porous ortho- and pyrophosphate-containing glass microspheres prepared by a sol–gel method provide very good physical characteristics not only for cell attachment and proliferation but also as a drug delivery system [11]. These glass microspheres can also be incorporated in 3D-printed matrices, e.g., from thermoplastic collagen, as described in another in vitro study in this Special Issue [12]. While this article provides valuable protocols to generate 3D-printed matrices from thermoplastic collagen, another study describing two novel bone adhesives focused more on characterizing their bond strength and adhesion mechanisms, which differ between natural and synthetic polymer adhesives [13].

Furthermore, other studies provide novel techniques to analyze fracture healing in vivo, e.g., multicolor histochemical staining for the identification of mineralized and non-mineralized musculoskeletal tissue in decalcified bone samples [14], or finite element model simulating three-point-bending tests in mouse tibiae [15]. The development of such techniques helps to apply the 3Rs principle (reduce, replace, refine) in in vivo studies.

Overall, this Special Issue provides a comprehensive overview of the latest advances in fracture healing research, highlighting the potential for new and innovative approaches to improve bone healing outcomes.
